# Mapping knowledge structure and research of the biologic treatment of asthma: A bibliometric study

**DOI:** 10.3389/fimmu.2023.1034755

**Published:** 2023-02-09

**Authors:** Jiamin Sun, Shiyao Bai, Jieyu Zhao, Danling Li, Xueqing Ma, Lin Ma, Xinming Su

**Affiliations:** Department of Pulmonary and Critical Care Medicine, Institute of Respiratory Diseases, The First Hospital of China Medical University, China Medical University, Shenyang, China

**Keywords:** asthma, biologics, treatment, visualization analysis, bibliometric analysis

## Abstract

**Background:**

Bronchial asthma (asthma) is a chronic inflammatory disease of the airways, involving a variety of cells and cellular components, that manifests clinically as recurrent episodes of wheezing, shortness of breath, with or without chest tightness or cough, airway hyperresponsiveness, and variable airflow limitation. The number of people with asthma has reached 358 million worldwide and asthma causes huge economic loss. However, there is a subset of patients who are not sensitive to existing drugs and the existing drugs have many adverse effects. Therefore, it’s important to find new drugs for asthma patients.

**Methods:**

Publications related to biologics in asthma published from 2000 to 2022 were retrieved from Web of Science Core Collection. The search strategies were as follows: topic: TS=(biologic* OR “biologic* product*” OR “biologic* therap*” OR biotherapy* OR “biologic* agent*” OR Benralizumab OR “MEDI-563” OR Fasenra OR “BIW-8405” OR Dupilumab OR SAR231893 OR “SAR-231893” OR Dupixent OR REGN668 OR “REGN-668” OR Mepolizumab OR Bosatria OR “SB-240563” OR SB240563 OR Nucala OR Omalizumab OR Xolair OR Reslizumab OR “SCH-55700” OR SCH55700 OR “CEP-38072” OR CEP38072 OR Cinqair OR “DCP-835” OR DCP835 OR Tezspire OR “tezepelumab-ekko” OR “AMG-157” OR tezspire OR “MEDI-9929” OR “MEDI-19929” OR MEDI9929 OR Itepekimab OR “REGN-3500”OR REGN3500 OR “SAR-440340”OR SAR440340 OR Tralokinumab OR “CAT-354” OR Anrukinzumab OR “IMA-638” OR Lebrikizumab OR “RO-5490255”OR “RG-3637”OR “TNX-650”OR “MILR1444A”OR “MILR-1444A”OR”PRO301444”OR “PRO-301444”OR Pitrakinra OR altrakincept OR “AMG-317”OR”AMG317” OR Etokimab OR Pascolizumab OR “IMA-026”OR Enokizumab OR “MEDI-528”OR “7F3COM-2H2” OR 7F3COM2H2 OR Brodalumab OR “KHK-4827” OR “KHK4827”OR “AMG-827”OR Siliq OR Ligelizumab OR “QGE-031” OR QGE031 OR Quilizumab OR Talizumab OR “TNX-901” OR TNX901 OR Infliximab OR Etanercept OR “PRS-060”) AND TS=asthma*. The document type was set to articles and review articles and the language restriction was set to English. Three different analysis tools including one online platform, VOS viewer1.6.18, and CiteSpace V 6.1.R1 software were used to conduct this bibliometric study.

**Results:**

This bibliometric study included 1,267 English papers published in 244 journals from 2,012 institutions in 69 countries/regions. Omalizumab, benralizumab, mepolizumab, and tezepelumab in relation to asthma were the research hotspots in the field.

**Conclusion:**

This study systematically uncovers a holistic picture of existing literature related to the biologic treatment of asthma over the past 20 years. We consulted scholars in order to understand key information in this field from the perspective of bibliometrics, which we believe may greatly facilitate future research in this field.

## 1 Introduction

Bronchial asthma (asthma) is a chronic inflammatory disease of the airways, involving a variety of cells and cellular components, that manifests clinically as recurrent episodes of wheezing, shortness of breath, with or without chest tightness or cough, airway hyperresponsiveness, and variable airflow limitation, which can lead to airway remodeling as the disease prolongs (1). The number of people with asthma has reached 358 million worldwide and the average prevalence of childhood and adult asthma in Asia has been on the rise in recent years (2, 3). Asthma affects people’s health, reduces quality of life, consumes a large amount of medical resources, and is a heavy economic burden on a country. Asthma is considered severe if it is uncontrolled despite adherence to maximally optimized high-dose ICS-LABA treatment and management of contributory factors or if it worsens when high-dose treatment is reduced (4). Biologic agents are therapeutics that are synthesized by living organisms and bind to a specific determinant. Because of this selectivity, biologic agents are ideal for ‘personalized’ or ‘precision’ medicine (5). Biologic agents targeting inflammatory cytokines, comprising anti-immunoglobulin (Ig)E and anti-interleukin (IL)-4/IL-13, IL-5, IL-5R, and thymic stromal lymphopoietin (TSLP) have emerged as promising treatment options for patients with severe asthma (6).

Bibliometrics are quantitative methods of studying scientific research using publications as a proxy for research. Bibliometrics enable and empower scholars to gain a one-stop overview, identify knowledge gaps, derive novel ideas for investigation, and propose their intended contributions to the field (7). Over the past 22 years, a vast body of studies has been published concerning biologic agents. Such significant growth in the literature requires new approaches to review and analyze trends.

Therefore, in this study, based on the Web of Science Core Collection (WOSCC) database, we used CiteSpace, VOSviewer, and R-Bibliometrix to conduct a bibliometric and visual analysis of the number of publications, citations, and research trends of countries/regions, authors, institutions, and keywords in literature related to the biologic treatment of asthma; identified the research hotspots; and predicted development trends in this field.

## 2 Materials and methods

### 2.1 Data source and search strategies

We comprehensively searched publications related to the biologic treatment of asthma in the WOSCC database from 2000 to 2022. All searches were completed and downloaded on the same day, 18 November 2022, to avoid any bias caused by daily database updates, and were verified by two authors (JS and SB) independently. The discrepancies were resolved by discussion with the corresponding author. The retrieval strategy was as follows: TS=(biologic* OR “biologic* product*” OR “biologic* therap*” OR biotherapy* OR “biologic* agent*” OR Benralizumab OR “MEDI-563” OR Fasenra OR “BIW-8405” OR Dupilumab OR SAR231893 OR “SAR-231893” OR Dupixent OR REGN668 OR “REGN-668” OR Mepolizumab OR Bosatria OR “SB-240563” OR SB240563 OR Nucala OR Omalizumab OR Xolair OR Reslizumab OR “SCH-55700” OR SCH55700 OR “CEP-38072” OR CEP38072 OR Cinqair OR “DCP-835” OR DCP835 OR Tezspire OR “tezepelumab-ekko” OR “AMG-157” OR tezspire OR “MEDI-9929” OR “MEDI-19929” OR MEDI9929 OR Itepekimab OR “REGN-3500”OR REGN3500 OR “SAR-440340”OR SAR440340 OR Tralokinumab OR “CAT-354” OR Anrukinzumab OR “IMA-638” OR Lebrikizumab OR “RO-5490255”OR “RG-3637”OR “TNX-650”OR “MILR1444A” OR “MILR-1444A”OR”PRO301444”OR “PRO-301444”OR Pitrakinra OR altrakincept OR “AMG-317” OR “AMG317” OR Etokimab OR Pascolizumab OR “IMA-026”OR Enokizumab OR “MEDI-528”OR “7F3COM-2H2”OR 7F3COM2H2 OR Brodalumab OR “KHK-4827” OR “KHK4827” OR “AMG-827”OR Siliq OR Ligelizumab OR “QGE-031” OR QGE031 OR Quilizumab OR Talizumab OR “TNX-901” OR TNX901 OR Infliximab OR Etanercept OR “PRS-060”) AND TS=asthma*. Timespan: 2000 to 2022. The selection criteria were as follows: (1) language: English; (2) document type: article or review article; and (3) timespan: 2000–2022. Initially, there were 10,512 documents, and 7,683 satisfied these criteria. After screening each of these titles and abstracts, 1,267 articles that exclusively targeted the topic were selected.

### 2.2 Data analysis

CiteSpace is knowledge visualization software developed by Dr. Chen from Drexel University. It was developed with Java, based on co-citation analysis. CiteSpace (6.1.R3) was used to analyze the selected literature, including co-citation analysis performed on countries/regions and institutions, dual-map overlay of citations, co-cited references analysis, and references with the strongest citation burst.

VOSviewer (1.6.18) can be used to build metrological maps of authors, journals, countries, or keywords based on co-occurrence data. It includes Network Visualization, Overlay Visualization, and Density Visualization, with the advantages of easy drawing and comprehensive images (8). In the visual map, different nodes represent authors, journals, and keywords, etc.; the node size indicates numbers or frequency; the thickness of the line represents the strength of the link; the colors of the nodes represent different clusters or times.

We used R software and R-Bibliometrix to generate the distribution map of high-frequency keywords over time.

We also used Pajek, a professional web analytics tool, to make the picture clearer. This bibliometric analysis is reported in accordance with the PRISMA 2020 statement.

## 3 Results

### 3.1 Annual growth trend

**Figure 1:**
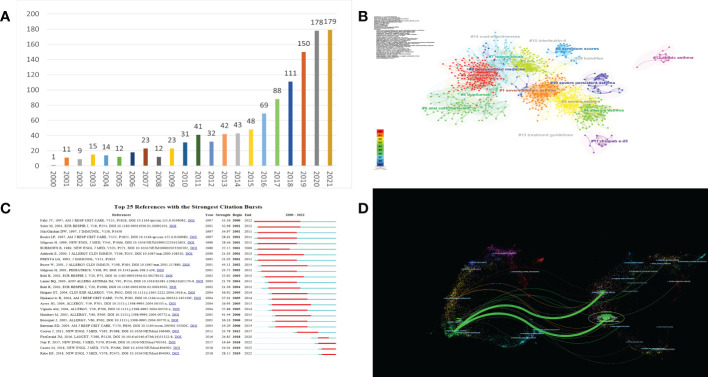
**(A)** Trends in the number of publications; **(B)** The co-citation clusters of references related to the biologic treatment of asthma; **(C)** Top 25 References with the strongest Citation Bursts; **(D)** A dual-map overlay of journals related to research on biologic the treatment of asthma.

The sample in this study is comprised of a total of 1,267 publications by 5,016 authors affiliated with 2,012 organizations in 69 countries, which were published in 244 journals. There were 941 articles (74.27%) and 326 reviews (25.73%) among the 1,267 documents. The distribution of publication numbers by year, presented in **Figure 1A**, shows a relatively upward trend, indicating steady development. From 2001 to 2008, the number of annual publications remained low. From 2008 to 2020, the number of annual publications began to grow rapidly, although there was a slight decline in volatility in 2012. This indicates that the application of biologic agents in asthma had begun to enter people’s vision and was attracting the attention of more scholars on a global scale.

### 3.2 Analysis of co-cited references: Clusters of research and most cited papers

We used CiteSpace software to generate a map of reference co-citations with corresponding clusters so that we could extract landmark references and clusters of research (**Figure 1B**). In this network of co-citation references, 80 different clusters were identified, and we filtered 17 clusters with significant modularity and silhouette scores (Q= 0.6713; S= 0.8708). Q-values range from 0-1, with values greater than 0.3 indicating a significant delineation structure; S-values greater than 0.5 indicating reasonable clustering results, and 0.7 or more being more convincing (9).

When two publications are cited jointly by a third publication, this is referred to as a co-citation relationship, and co-cited references are usually regarded as a knowledge base in a particular field (9). In the visualization network of co-cited references, all nodes representing the references were clustered into 17 specific clusters with the highest K values, including “#0 omalizumab”, “#1 gemcitabine”, “#2 severe allergic asthma”, “# 3 il-5”, and so on.

A citation burst provides evidence that a particular publication is associated with a surge of citations, which indicates that the publication has evidently attracted the attention of its scientific community. As shown in **Figure 1C**, since 2000, the analysis of burstiness revealed that the top three references with the strongest citation bursts are “Omalizumab, anti-IgE recombinant humanized monoclonal antibody, for the treatment of severe allergic asthma”, “The anti-IgE antibody omalizumab reduces exacerbations and steroid requirement in allergic asthmatics”, and “Benefits of omalizumab as add-on therapy in patients with severe persistent asthma who are inadequately controlled despite best available therapy” (GINA 2002 step 4 treatment): INNOVATE.

### 3.3 Journals and co-cited academic journals analysis

244 journals published 1,267 publications regarding the biologic treatment of asthma, and 55 journals published more than five publications. Among the top 10 journals in terms of the number of papers, Journal of Asthma is ranked first (64 papers), followed by Journal of Asthma and Journal of Allergy and Clinical Immunology-In Practice (62 and 55 papers, respectively). The top 10 most-cited journals are listed in **Table 1**. The influence of a journal depends on the number of times it is co-cited, which reflects whether the journal has essential influence in a specific field (10). Thirteen journals had been cited more than 1000 times, and the journal with the highest number of citations is the Journal of Allergy and Clinical Immunology (7,043), followed by the American Journal of Respiratory and Critical Care Medicine (3, 7, 11).

**Table 1:** The top 10 journals with the most publications.

source	documents	citations	Average citation/publication	IF (2021)	JCR (2021)	Country
Journal of Asthma	64	1041	16.27	2.5149	Q4	United States
Journal of Allergy And Clinical Immunology-In Practice	62	1317	21.24	11.0226	Q1	Netherlands
annals of allergy asthma and immunology	55	1193	21.69	1.5702	Q4	Iran
journal of allergy and clinical immunology	46	6198	134.74	14.2905	Q1	United States
respiratory medicine	45	1905	42.33	4.5817	Q2	England
allergy	43	3539	82.30	14.7097	Q1	England
journal of asthma and allergy	39	337	8.64	3.0272	Q3/Q4	New Zealand
expert opinion on biological therapy	28	461	16.46	5.5886	Q1/Q2	England
allergy and asthma proceedings	26	1654	63.62	2.8773	Q4	United States
clinical and experimental allergy	26	309	11.88	5.4008	Q2	England

The dual-map overlay of journals represents the scientific distribution of academic journals (**Figure 1D**). The citing journals are located on the left side, while the cited journals are on the right side, and the colored paths illustrate the citation relationships, indicating the citation trajectory and flow of knowledge (12). As shown, the result indicates that the citing papers are mainly focused on journals in the fields of medicine, medical, and clinical, whereas most of the cited articles were published in journals in the fields of health, nursing, molecular, biology, genetics, and medicine.

### 3.4 Authors analysis

The top five productive authors are Busse, William W. (n=31), Canonica, Giorgio Walter (n=31), Pelaia, Corrado (n=29), Pavord, Ian D. (n=29), and Corren, Jonathan (n=27). Although Pavord, Ian D. and Pelaia, Corrado are tied for second in the number of publications, the total and average number of citations of Pavord, Ian D. is ranked first (5,926 and 204.35), which suggests he has a significant influential status in this field.

**Figure 2:**
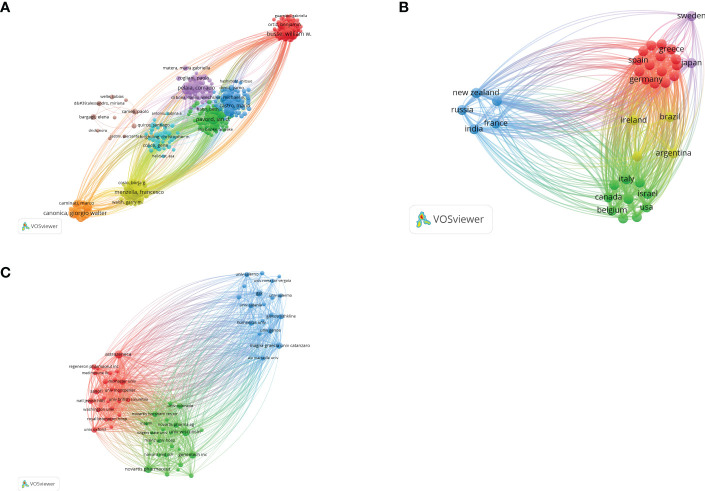
**(A)** The visualization map of collaborations between core authors. The lines between nodes represent cooperation between different authors. Each node represents one author. The size of the node is proportional to the number of documents published. **(B)** The cross-country collaboration visualization map. The lines between nodes represent cooperation between countries. Each node represents a country or region. The size of the node is proportional to the number of documents published; **(C)** The visualization map of collaborations between institutions. The lines between nodes represent cooperation between institutions. Each node represents one institution. The size of the node is proportional to the number of documents published.

According to Price’s Law, the minimum number of core authors in a field is m=0.749× $\sqrt{n}$ . ,and n is the maximum number of authors: n=29, m≈4.03. Therefore, authors with more than four publications are identified as core authors in this field. There are 351 core authors with ≥4 publications. **Figure 2A** shows the map of author co-authorship analysis. Only 351 core authors with ≥4 papers were included, forming a total of eight author clusters. By analyzing the co-citation network of authors, 153 authors who had been cited more than 50 times were divided into three author clusters (**Figure 2A**). The connection represents the cooperation between authors, and the size of the circle represents the number of citations. Total link strength (TLS) indicates the impact of authors’ published papers on other authors involved in the studies. Castro, M has the greatest TLS (n =23,684), followed by Corren, J. (n = 21,126) and Hanania, Na (n = 15,808).

### 3.5 Countries/regions analysis

According to the VOSviewer analysis, a total of 1,267 articles are from 69 countries. As shown in **Figure 2B**, the top five countries/regions are the United States (n =514, 40.57%), England (n =235, 18.55%), Italy (n =214, 16.89%), France (n =122, 9.62%), and Canada (n =120, 9.47%). Strikingly, the top five countries contributed 95.10% of the total number of publications. Moreover, as illustrated in **Figure 2B**, each node represents a country or region, and the size of the node is proportional to the number of papers published. The lines between nodes represent cooperation between countries. We found that the most obvious cooperation is between England and the United States. The total link strength of the United States is significantly higher than that of other countries, indicating that it cooperates more closely with other countries. This is also reflected in the fact that European and American countries have a large number of articles, while Asian, African, and Latin American countries have a small number of articles, as shown in **Figure 2B**.

### 3.6 Institutions analysis

These documents were contributed by 2,012 institutions, with the top 10 institutions contributing a total of 415 articles, accounting for 32.75% of the total (**Figure 2C**). As shown in **Figure 2C**, the top three institutions/affiliations with the most documents are AstraZeneca (n=57), University of Wisconsin-Madison (n = 57), and Novartis (n = 54). Remarkably, six of the top 10 institutions are pharmaceutical companies; for example, Novartis is one of the three largest pharmaceutical companies in the world and developed omalizumab, the world’s first biologic drug for the treatment of asthma.

### 3.7 Keyword analysis

**Figure 3:**
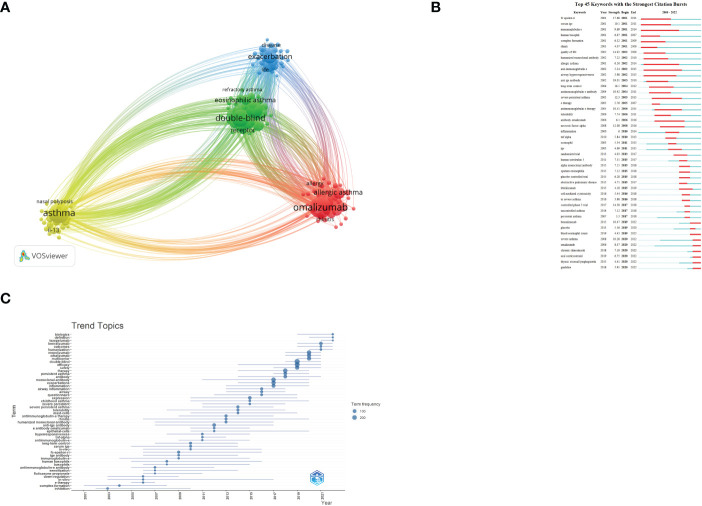
**(A)** Network visualization of keywords; **(B)** Top 45 keywords with strongest Citation Bursts; **(C)** Trend topics. The X-axis represents the year, while the Y-axis is the cumulative occurrences of the keywords.

**Figure 3A** shows the network visualization of keywords generated by VOSviewer. Among 2,424 keywords, the frequency of occurrence was set to at least 20, and finally, 119 keywords were included in the analysis. The keywords appearing at a high frequency within these documents are omalizumab (526), asthma (466), double-blind (346), mepolizumab (334), efficacy (292), severe asthma (238), exacerbation (234), monoclonal-antibody (212), safety (205), benralizumab (191), and so on. **Figure 3A** shows the blue, red, green, and yellow clusters, representing four research clusters. CiteSpace was also used to illustrate the keywords with strong citation bursts. Burst Terms refer to words with large changes in word frequency in a short period of time, which can reflect the research frontier to a certain extent. As **Figure 3B** demonstrates, we screened out the top seven keywords whose citation bursts continued to 2022, including “benralizumab”, “blood eosinophil count”, “severe asthma”, “omalizumab”, “chronic rhinosinusitis”, “oral corticosteroid”, “thymic stromal lymphopoietin”, and “guideline”. The trend topic analysis was an important mapping tool that helped to portray the seed of trend integration rooted in the previous stream (13). The trend topics map was generated by R-Bibliometrix based on the occurrence frequency of author keywords and set the word minimum frequency at five and the number of words per year at three (**Figure 3C**). The results show that “Tezepelumab”, “humanization”, “definition”, “multicenter”, “benralizumab”, “outcomes”, and “biologics” are the words with the highest occurrence frequency in the past three years.

## 4 Discussion

In this study, we used CiteSpace, VOSviewer, and R-Bibliometrix to conduct a bibliometric and visual analysis of 1,267 publications related to the biologic treatment of asthma published between 2000 and 2022 (as of 18 November), to identify the research status of global publications related to this field, summarize research hotspots, and predict future research trends.

From 2000 to 2022, publications related to the biologic treatment of asthma have shown a significant growth trend. From 2001 to 2007, the number of publications related to the biologic treatment of asthma was still small and the trend was unstable. From 2008 to 2021, the number of publications increased steadily, and the annual number of publications has exceeded 100 since 2018, although there was a slight decline in 2012. These results show that between 2012 and 2022, research on the biologic treatment of asthma began to develop rapidly.

The number of publications in a country is an important indicator of a country’s academic ability. **Figure 2B** shows that the top five countries, i.e., the United States, England, Italy, France, and Canada, contributed 95.10% of the total number of publications. This shows that European and American countries have produced more in-depth studies in this area. The United States has the highest total number of citations and total link strength. These results reflect that the United States has made great contributions and established its leading position in the field. As for cooperation between countries, it can be seen from **Figure 2B** that, among the 20 countries with the most publications, the United States and England have the closest cooperation with other countries/regions; the United States and the European countries (e.g., Italy, England, and France) are the key players in the international cooperation landscape, and extensive cooperation has been established between them.

Among the top five authors who published the most papers, Pavord, Ian D. and Pelaia, Corrado are tied for second place in the number of publications, yet the total and average number of citations of Pavord, Ian D. is ranked first (5,926 and 204.35 respectively). As can be seen among the top five authors, Busse, William W. and Corren, Jonathan carried out research in this field first, starting in 2001, when Busse, William W. published a paper titled “Anti-immunoglobulin E (omalizumab) therapy in allergic asthma”. This paper shows that omalizumab can reduce asthma exacerbations and improve quality of life and that omalizumab is safe and well tolerated (14). In the same year, Corren, Jonathan published an article called “Omalizumab, anti-IgE recombinant humanized monoclonal antibody, for the treatment of severe allergic asthma”. This paper shows that the addition of omalizumab to standard asthma therapy reduces asthma exacerbations and decreases inhaled corticosteroid and rescue medication use (15). In 2007, Pavord, Ian D. carried out research in this field and published multiple articles in The New England Journal of Medicine, The Lancet, and The Lancet Respiratory Medicine. The research of Pavord, Ian D. focuses on mepolizumab and shows that mepolizumab is an effective and well-tolerated treatment that reduces exacerbations and improves AQLQ scores in patients with refractory eosinophilic asthma (11, 16, 17), and baseline eosinophil thresholds may help to select patients who are likely to experience important asthma outcomes with mepolizumab (18).

Analyzing the characteristics of international peer-reviewed journals is helpful to understand the current trend, which directly helps scholars identify the important journals related to their fields and select the most appropriate published journals for their research (13). As shown in **Tables 1**, **2**, Allergy, Clinical and Experimental Allergy, Journal of Allergy and Clinical Immunology, and Respiratory Medicine are not only in the top 10 journals with the most publications but also the top 10 co-cited journals ranked by number of citations, and the four journals are all JCR Q1 or Q2 journals, which shows that they are important journals in this field. It is necessary to pay attention to the published papers in these journals to obtain the latest advances in this field.

**Table 2:** The top 10 journals.

source	citations	IF (2021)	JCR (2021)	Country
Journal of Allergy and Clinical Immunology	7043	14.2903	Q1	United States
American Journal of Respiratory and Critical Care Medicine	3716	30.5293	Q1	United States
New England Journal of Medicine	3319	176.0774	Q1	United States
European Respiratory Journal	3115	33.7954	Q1	England
Allergy	2540	14.7097	Q1	England
Lancet	1906	202.7275	Q1	England
Respiratory Medicine	1491	4.5817	Q2	England
Lancet Respiratory Medicine	1325	102.6413	Q1	England
Clinical and Experimental Allergy	1264	5.4008	Q2	England
Chest	1173	10.2662	Q1	United States

**Figure 4:**
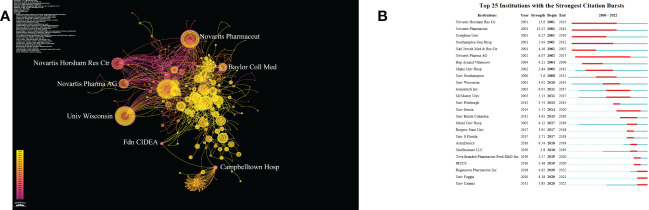
**(A)** The cooperation network of institutions generated by CiteSpace. In the visualization map, one node represents an institution, and its size isproportional to the number of publications. The links between nodes represent the strength of cooperation. **(B)** Top 25 Institutions with strongest Citation Bursts.

Betweenness Centrality measures the probability that a point is in the shortest path between any other two points. This concept was proposed by Linton C. Freeman in 1977, which can effectively calculate the points that play the important roles of bridge and medium between multiple parts of a graph. The value range of mediation centrality is [0,1], the higher the value, the stronger the mediation effect (19, 20). As **Figure 4A** shows, Novartis Pharmaceuticals Corporation, Novartis Horsham Research Centre, Baylor College of Medicine, Novartis International AG, University of Wisconsin–Madison, Campbelltown Hospital, and Fdn CIDEA have BC values greater than 0.1 (0.21, 0.13, 0.15, 0.14, 0.12, 0.13, and 0.12 respectively). This indicates that these seven institutions play an important role as a bridge in the communication and cooperation between different institutions. Novartis Pharmaceuticals Corporation and the University of Wisconsin–Madison are not only in the top 10 institutions by publications but also have high BC values, indicating that they are at the core of international cooperation in this field. As **Figure 4B** demonstrates, we screened out the top three institutions whose citation bursts continued to 2022, including “Regeneron Pharmaceuticals Inc”, “University of Foggia”, and “University of Catania”, indicating these three institutions may become influential institutions in this field in the future.

As **Figure 3B** demonstrates, we screened out the top four keywords whose citation bursts continued to 2022, including “benralizumab”, “severe asthma”, “blood eosinophil count”, “omalizumab”, “rhinosinusitis”, “oral corticosteroid”, “thymic stromal lymphopoietin”, and “guideline”. In addition, we also conducted a trend topic analysis based on author keywords (**Figure 3C**), and the results show that “mepolizumab”, “omalizumab”, and “multicenter” began to appear in the field in 2020, and “benralizumab”, “outcomes”, and “humanization” began to appear in the field in 2021.”Biologics”, “definition”, and “tezepelumab” began to appear in 2022; researchers gradually paid increasing attention to these keywords. Using the following three topics, we will analyze the research status of biologic agents in asthma.

### 4.1 Omalizumab and asthma

In type 2-high asthma, B cells produce IgE under the influence of the Th2 cytokines, IL-4 and IL-13. IgE can bind to the high-affinity Fcϵ RI on the surface of basophils, mast cells, eosinophils, and DCs. This leads to immediate degranulation and the release of many mediators associated with asthma (21). Omalizumab selectively targets human immunoglobulin (Ig)E, forming small immune complexes that inhibit IgE from binding to its high- and low-affinity receptors. Therefore, omalizumab effectively blunts the immune response in asthmatic patients, significantly improving control over asthma symptoms and successfully preventing disease exacerbations (22). Omalizumab was the first available monoclonal antibody for the add-on treatment of severe allergic asthma. Omalizumab has reduced the annualized rate of severe exacerbations, symptoms, oral corticosteroid doses, associated work/school days lost, and healthcare resource utilization and improves quality of life in patients with severe allergic asthma (23, 24). Omalizumab is applicable for patients aged ≥6 years with moderate or severe allergic asthma that is uncontrolled with Step 4–5 treatment (25). The Xolair Pregnancy Registry (EXPECT) shows that there is no evidence that omalizumab increases the risk of major congenital anomalies, prematurity, low birth weight, and small size for gestational age during pregnancy (26). Chronic rhinosinusitis with nasal polyps (CRSwNP) and asthma are common comorbidities and share similar pathophysiology. Clinically, asthma in the presence of nasal polyposis is more difficult to control, being more exacerbation prone, with increased airway obstruction and more extensive eosinophilic inflammation (27). Add-on omalizumab for severe allergic asthma is effective in improving asthma control and lung function and reducing exacerbations, including in asthma patients with CRSwNP (28). Clinical studies have shown that the most common adverse effects in relation to omalizumab are arthralgia, pain (general), leg pain, fatigue, vertigo, fractures, arm pain, pruritus, dermatitis, and earache.

### 4.2 Benralizumab and mepolizumab in relation to asthma

Eosinophils represent a proinflammatory granulocyte that plays a major role in type 2-high asthma. IL-5 is a homodimeric cytokine mainly secreted by activated T helper lymphocyte type 2 cells, mast cells, and innate lymphoid cells, and the differentiation, activation, and survival of eosinophils is driven by IL-5 (21). Therefore, inhibiting IL-5 or IL-5 signaling seems to be a good option for asthma. Mepolizumab binds to IL-5, thus preventing it from binding to IL-5R, and benralizumab targets interleukin-5 receptor α, which depletes eosinophils by antibody-dependent cell-mediated cytotoxicity. Mepolizumab and benralizumab are licensed for the treatment of severe eosinophilic asthma (SEA).

Benralizumab is a humanized, afucosylated, monoclonal antibody, which targets IL-5 receptor α and induces the direct, rapid, and almost complete depletion of eosinophils through enhanced antibody-dependent cell-mediated cytotoxicity, an apoptotic process of eosinophil elimination involving natural killer cells (29). Benralizumab significantly reduces airway wall and sputum eosinophil and is applicable for patients with mild to severe asthma. It significantly reduces asthma exacerbations, improves lung function and quality of life, and reduces oral corticosteroid doses for patients with severe eosinophilic asthma, with treatment effects sustained for up to two years (30, 31). Furthermore, benralizumab can represent a valid add-on therapeutic option for patients with severe eosinophilic asthma, especially with comorbid CRSwNP (32). Clinical studies have shown that the most common mild or moderate adverse events related to benralizumab are nasopharyngitis, headaches, sinusitis, bronchitis, and pyrexia; the most common adverse event is worsening of asthma; and the serious adverse events related to treatment are cytokine release syndrome, mydriasis, pneumonia, urticaria, allergic granulomatous angiitis, panic attacks, paresthesia, and so on (29, 31).

Mepolizumab is effective at eosinophil values of 150 cells/µL and above. Several clinical trials have shown the efficacy of mepolizumab in reducing exacerbations and oral corticosteroid use and in the improvement of quality of life, asthma control, and lung function (33). Patients who stopped mepolizumab experienced an increase in exacerbations and reduced asthma control versus those who continued (34). Mepolizumab improves sino-nasal and asthma symptoms and reduces polyp growth in patients with severe eosinophilic asthma and concomitant CRSwNP (35). Clinical trials have shown that the most frequently reported adverse events in relation to mepolizumab are headaches and nasopharyngitis and the most frequently reported drug-related adverse event is infusion-related reactions (16, 17). There is no published RCT on pregnancy exposure to mepolizumab and benralizumab (36).

Receive subcutaneous mepolizumab in patients aged ≥6 years with severe eosinophilic asthma that is uncontrolled on Step 4–5 treatment and receive subcutaneous benralizumab for those aged ≥12 years, with severe eosinophilic asthma that is uncontrolled on Step 4–5 treatment (25).

### 4.3 Tezepelumab and asthma

TSLP is an epithelial-cell-derived cytokine, belonging to the alarmins group, which plays a key pathogenic role in asthma by acting as an upstream activator of cellular and molecular pathways leading to type 2 (T2-high) airway inflammation (37). Tezepelumab is a human monoclonal antibody (IgG2λ) that blocks thymic stromal lymphopoietin, inhibiting the production of multiple inflammatory cytokines and the activation of multiple cell types (6). Studies have shown that tezepelumab reduces type 2 (T2) inflammatory biomarker levels and oral corticosteroid (OCS) doses. Furthermore, patients with severe, uncontrolled asthma, with or without perennial allergy, including those with low blood eosinophil counts (<300 cells per microliter) at baseline who received tezepelumab in relevant clinical trials had fewer exacerbations and better lung function, asthma control, and health-related quality of life than those who received placebo (28, 38–41). The most common adverse events reported in relation to tezepelumab are nasopharyngitis, upper respiratory tract infections, headaches, and asthma­related events, but the frequencies and types of adverse events did not differ meaningfully between the tezepelumab and placebo groups in the relevant studies. The serious adverse events reported are pneumonia, stroke, and Guillain–Barré syndrome. There are no reported treatment-related anaphylactic reactions (38, 42). There is little literature on the treatment of asthma and comorbidities with tezepelumab, and there are no available published human data on tezepelumab exposure during pregnancy to evaluate any drug-associated risk of adverse maternal and fetal outcomes (36). Receive subcutaneous tezepelumab in patients aged ≥12 years with severe asthma (25).

## 5 Limitations

There are some limitations to the research. Firstly, the publications included in the analysis had a cut-off date of 18 November 2022, but the WOS Core Collection data continues to be updated and some of the 2022 literature is already online, which was not included in our work, so our paper does not fully reflect the reality of the situation in 2022. Secondly, only English documents were included; thus, contributions in other languages may have been overlooked. Third, because of the format requirement of CiteSpace and Vosviwer, we were unable to merge multiple databases for analysis, which may mean that articles that are only in other databases were ignored, such as PubMed, Embase, and Scopus. However, because of the significant cross-replication of the literature in the various databases and the authority of the WOSCC database, we believe that this work can still be applied to present the overall situation and general trend in this field.

## 6 Conclusion

This study was undertaken to systematically summarize the knowledge structure and research frontiers regarding the biologic treatment of asthma by means of bibliometric and visualization analysis, providing contemporary and future perspectives. The number of publications in the field has increased year by year, indicating that research related to this field has attracted significant interest in recent years. The current research focuses on omalizumab, benralizumab, mepolizumab, and tezepelumab in relation to asthma. Our study outlines basic scientific knowledge and various interrelationships concerning biologic treatment and also provides essential insights into research trends and frontiers. It is our hope that this study will help researchers to better grasp current overall trends in this field.

## Data availability statement

The original contributions presented in the study are included in the article/supplementary material. Further inquiries can be directed to the corresponding author.

## Author contributions

JS and XS conceived the study and its design. JS and SB collected the data and JS, SB, JZ, DL, XM and LM managed, analyzed, and interpreted the data. All authors contributed to the article and approved the submitted version.
