# Corrigendum: Mapping knowledge structure and research of the biologic treatment of asthma: a bibliometric study

**DOI:** 10.3389/fimmu.2025.1630817

**Published:** 2025-07-08

**Authors:** Jiamin Sun, Shiyao Bai, Jieyu Zhao, Danling Li, Xueqing Ma, Lin Ma, Xinming Su

**Affiliations:** Department of Pulmonary and Critical Care Medicine, Institute of Respiratory Diseases, The First Hospital of China Medical University, China Medical University, Shenyang, China

**Keywords:** asthma, biologics, treatment, visualization analysis, bibliometric analysis

In the published article, there was an error in **Figure 2** as published. In the country/region analysis section, when we used software to draw the knowledge map, we obtained several different versions of pictures. They had different focuses. The picture presented in the article makes the cooperative relationships between different countries and regions clearer. Because the volume of some representative country’s dots and some lines connecting closely cooperating countries is too large, it blocks some connections between countries. However, it fails to present some important aspects: for instance, the number of papers published by each country and the degree of collaboration between each country are different. And this will also cause confusion among readers as to why the points representing different countries and the lines representing different country partnerships end up being of the same size. The corrected **Figure 2** and its caption appear below.

**Figure 2:**
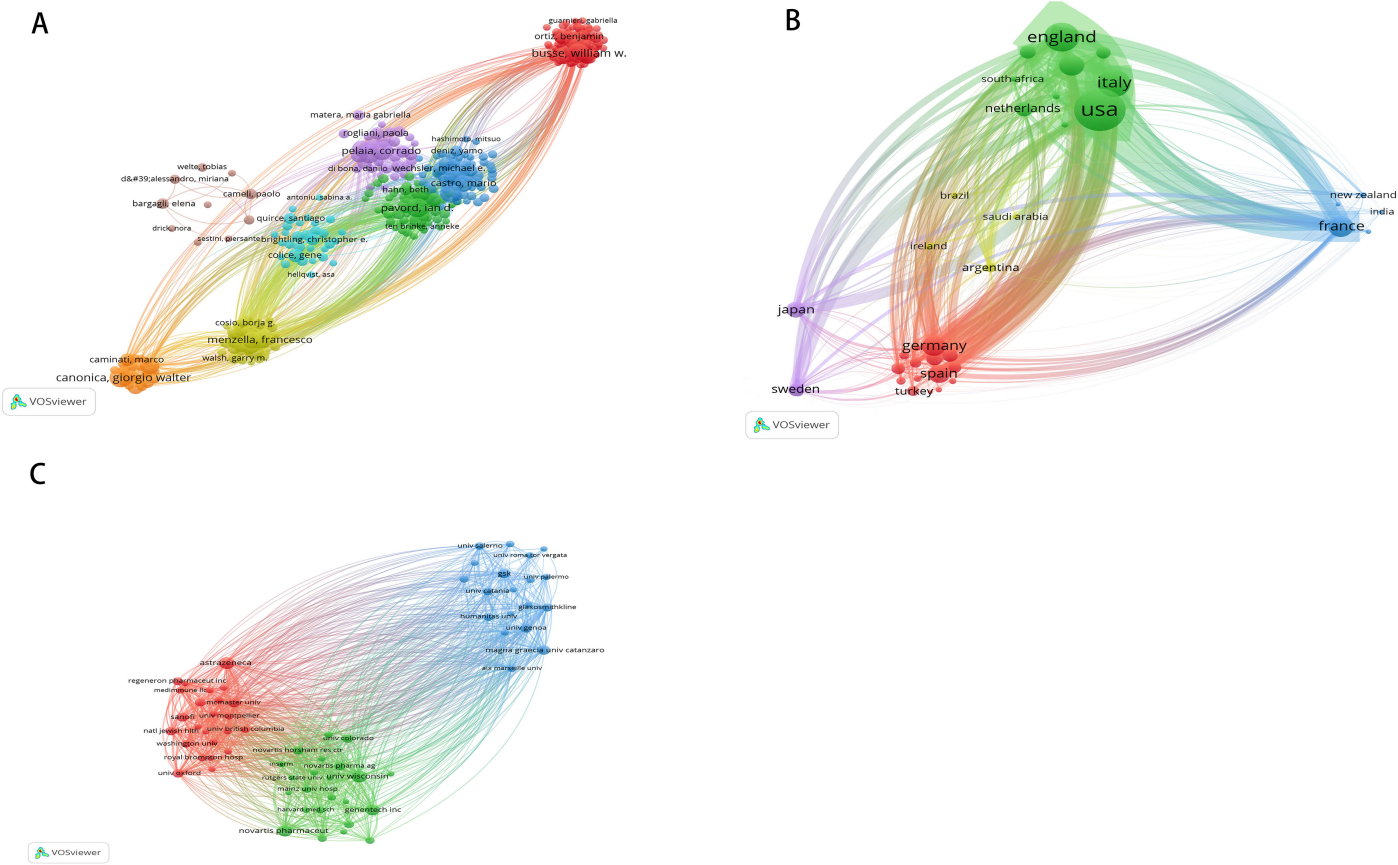
**(A)** The visualization map of collaborations between core authors. The lines between nodes represent cooperation between different authors. Each node represents one author. The size of the node is proportional to the number of documents published. **(B)** The cross-country collaboration visualization map. The lines between nodes represent cooperation between countries. Each node represents a country or region. The size of the node is proportional to the number of documents published; **(C)** The visualization map of collaborations between institutions. The lines between nodes represent cooperation between institutions. Each node represents one institution. The size of the node is proportional to the number of documents published.

The authors apologize for this error and state that this does not change the scientific conclusions of the article in any way. The original article has been updated.

In the published article, there was an error. Due to our negligence, there is inconsistency between the descriptions in the main text and those in the tables.

A correction has been made to **3 Results,** *3.3 Journals and co-cited academic journals analysis*, paragraph 1. This sentence previously stated:

“Among the top 10 journals in terms of the number of papers, Journal of Asthma is ranked first (64 papers), followed by Journal of Asthma and Journal of Allergy and Clinical Immunology-In Practice (62 and 55 papers, respectively).”

The corrected sentence appears below:

“Among the top 10 journals in terms of the number of papers, Journal of Asthma is ranked first (64 papers), followed by Journal of Allergy and Clinical Immunology-In Practice (62 and 55 papers, respectively).”

In the published article, there was an error. Due to our negligence, there was an error in substituting the value of n in the formula. A correction has been made to **3 Results**, *3.4 Authors analysis*, paragraph 2. This sentence previously stated:

“According to Price’s Law, the minimum number of core authors in a field is m=0.749× √n.,and n is the maximum number of authors: n=29, m≈4.03.”

The corrected sentence appears below:

“According to Price’s Law, the minimum number of core authors in a field is m=0.749× √n.,and n is the maximum number of authors: n=31, m≈4.17.”

In the published article, there was an error. In our initial version, regarding the section on authors analysis, apart from discussing and drawing the knowledge graph of the core authors, we also discussed and drew the knowledge graph of the authors with a high number of citations. However, after further discussion, we focused more on the analysis of the frontiers and hotspots of asthma. And due to the limitations of the number of figures and tables in the article, this part seemed overly redundant. Therefore, in the subsequent article versions, we deleted this figure and the related discussions. However, due to our oversight, this sentence was not completely deleted, causing confusion.

A correction has been made to **3 Results,** *3. 4 Authors analysis*, paragraph 2. This sentence previously stated:

“Only 351 core authors with ≥4 papers were included, forming a total of eight author clusters. By analyzing the co-citation network of authors, 153 authors who had been cited more than 50 times were divided into three author clusters (**Figure 2A**).”

The corrected sentence appears below:

“Only 351 core authors with ≥4 papers were included, forming a total of eight author clusters.”

The authors apologize for this error and state that this does not change the scientific conclusions of the article in any way. The original article has been updated.

In the published article, there was an error. We carefully read this text and discovered a small mistake.

A correction has been made to **3 Results**, *3.4 Authors analysis*, paragraph 2. This sentence previously stated:

“The connection represents the cooperation between authors, and the size of the circle represents the number of citations.”

The corrected sentence appears below:

“The connection represents the cooperation between authors, and the size of the circle represents the number of documents.”

The authors apologize for these errors and state that they do not change the scientific conclusions of the article in any way. The original article has been updated.

